# Blunt trauma to the antecubital fossa causing brachial artery injury and minor fractures around the elbow joint, an easily missed diagnosis with potential devastating consequences: a case report

**DOI:** 10.1186/s13256-018-1751-7

**Published:** 2018-07-18

**Authors:** Zi Hao Phang, Mohd Fadhli B. Miskon, Sa’adon B. Ibrahim

**Affiliations:** Hospital Sultan Ismail, Jalan Persiaran Mutiara Emas, Tmn Mount Austin, 81100 Johor Bahru, Malaysia

**Keywords:** Arterial injury, Blunt trauma, Brachial artery, Elbow dislocation, Vascular

## Abstract

**Background:**

Blunt trauma causing brachial artery injury in a young patient is very rare. Cases of brachial artery injury may be associated with closed elbow dislocation or instability. Elbow dislocation may not be evident clinically and radiologically on initial presentation.

**Case presentation:**

This is a case of a 37-year-old, right-hand dominant, Malay man who fell approximately 6 meters from a rambutan tree and his left arm hit the tree trunk on his way down. He was an active tobacco smoker with a 20 pack year smoking history. On clinical examination, Doppler signals over his radial and ulnar arteries were poor. He proceeded with emergency computed tomography angiogram of his left upper limb which showed non-opacification of contrast at the distal left brachial artery just before the bifurcation of the left brachial artery at his left elbow joint. Radiographs and computed tomography scan also showed undisplaced fracture of left lateral epicondyle and radial head with no evidence of elbow dislocation. He subsequently underwent left brachial to brachial artery bypass which was done using reversed saphenous vein graft and recovered well. His fractures were treated using 90 degree long posterior splint for 2 weeks and he was then allowed early range of motion of the left elbow. This patient developed left elbow dislocation 6 weeks postoperatively. Closed manipulative reduction of his left elbow resulted in incomplete reduction. The functional outcome of his left elbow was limited with a range of motion of left elbow of 0–45 degrees. However, he was not keen for surgery to stabilize his elbow joint during his last follow-up 6 months post injury.

**Conclusions:**

This is an uncommon case of brachial artery injury in a civilian caused by blunt trauma associated with occult elbow instability/dislocation and minor fractures around the elbow joint. The treatment of brachial artery injury with clinical evidence of distal ischemia is surgical revascularization. The possibility of elbow instability and dislocation need to be considered in all cases of brachial artery injury because early radiographs and computed tomography scans may be normal. Short-term posterior splint immobilization is not sufficient to prevent recurrent dislocations.

## Background

Arterial injury to the upper extremity is commonly caused by penetrating injuries [1]. Our understanding of the mechanisms of penetrating arterial injuries to an upper limb in military personnel dates back to World War II and was documented by DeBakey and Simeone [1]. However, the mechanism of low energy blunt trauma causing arterial injury to an upper limb in civilians is not well elucidated. Based on studies, only 6–20% of upper limb arterial injuries are caused by blunt trauma [2, 3]. These cases are often associated with either shoulder or elbow joint dislocations and are more prevalent in the older population [4, 5]. Older patients are presumably more prone to arterial injury due to loss of elasticity of their arteries. This is a case report of upper limb arterial injury caused by blunt trauma in a young patient associated with occult elbow instability and dislocation not evident clinically and radiologically. The primary aim of this report is to highlight that in a case of blunt trauma causing brachial artery injury, a normal computed tomography scan or radiograph does not rule out elbow dislocation or instability. A high index of suspicion is required to diagnose this condition. In addition, we conducted a literature review to discuss the current trend of management for elbow instability or dislocation associated with brachial artery injury.

## Case presentation

This is a case of a 37-year-old, right-hand dominant, Malay man who presented to our Emergency Department 6 hours after he had fallen approximately 6 meters from a rambutan tree where his left arm hit the tree trunk on his way down to the ground. Post trauma, he complained of pain and swelling over his left antecubital fossa. There was no wound over his left upper limb. He had no history of trauma to his left upper limb and no significant past medical history. He did not take any medications. He was an army officer and had been an army officer for 16 years. Two years prior to the current accident, he was transferred to the administration unit of the Ministry of Defense. His job scope was mainly office work. He lived with his wife and three children in a small suburban home. He was an active tobacco smoker with a 20 pack year smoking history. Currently he smoked 10–15 cigarettes a day. He did not consume alcohol.

**Fig. 1:**
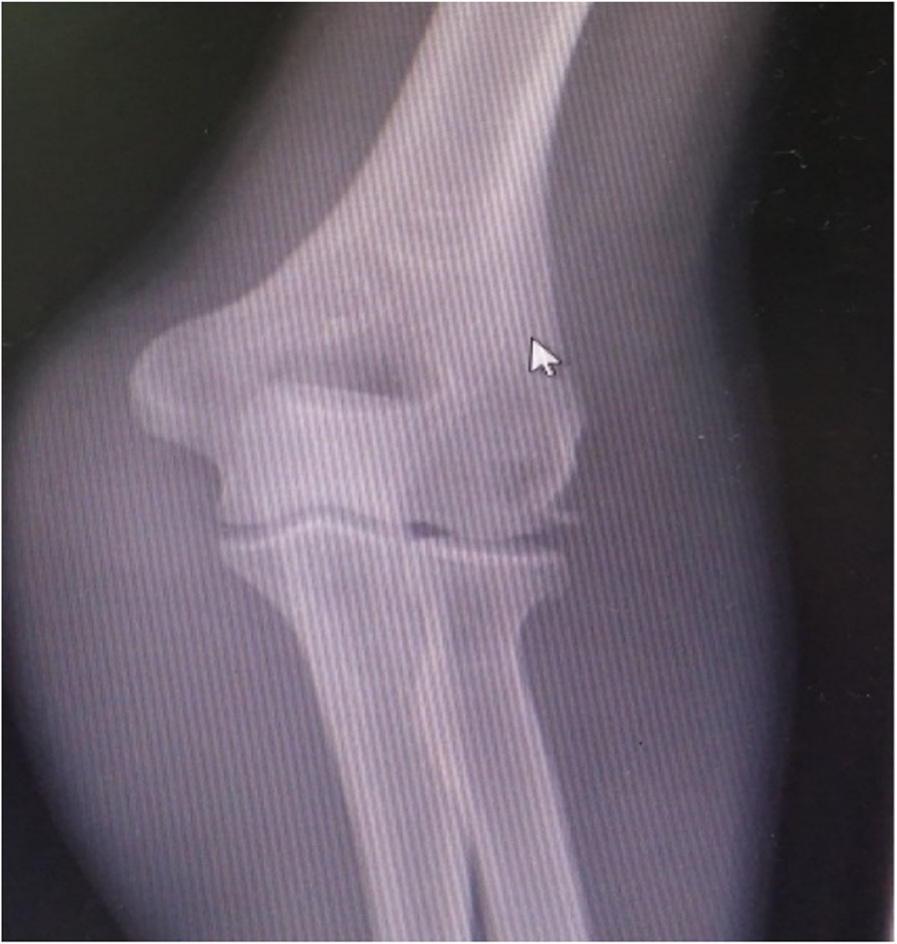
Anteroposterior view radiograph of left elbow. Arrow indicating fracture lateral epicondyle

**Fig. 2:**
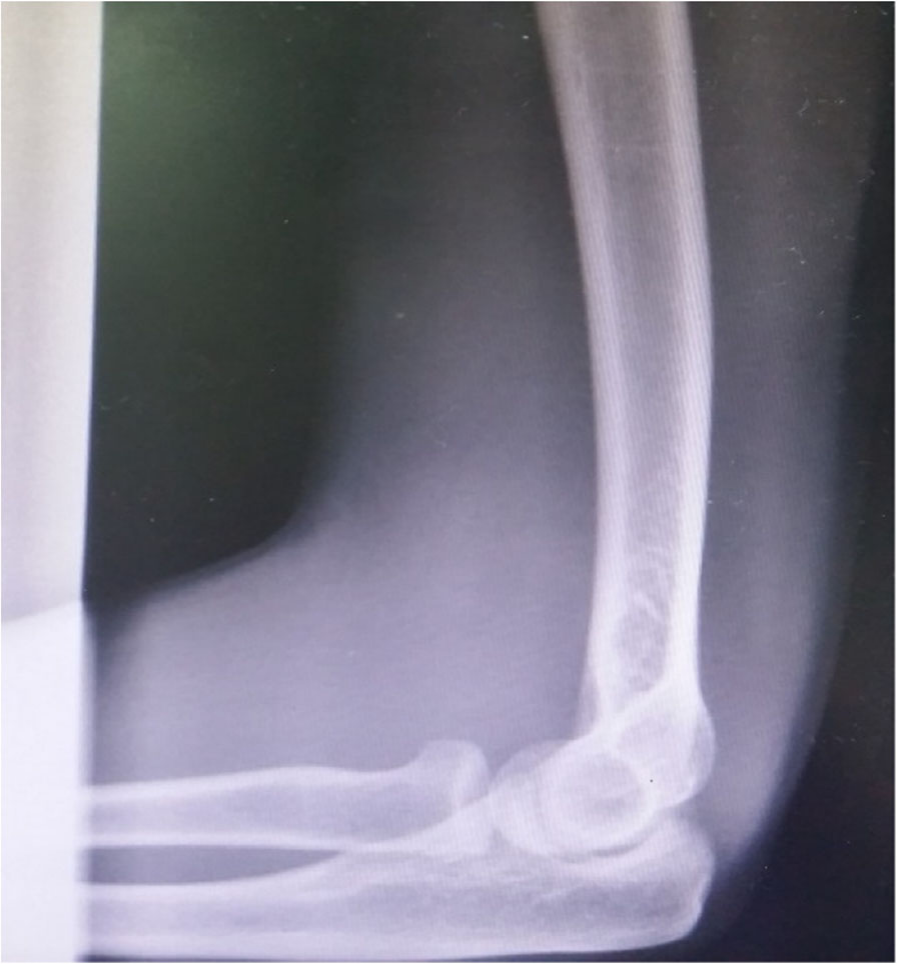
Lateral view radiograph of left elbow

**Fig. 3:**
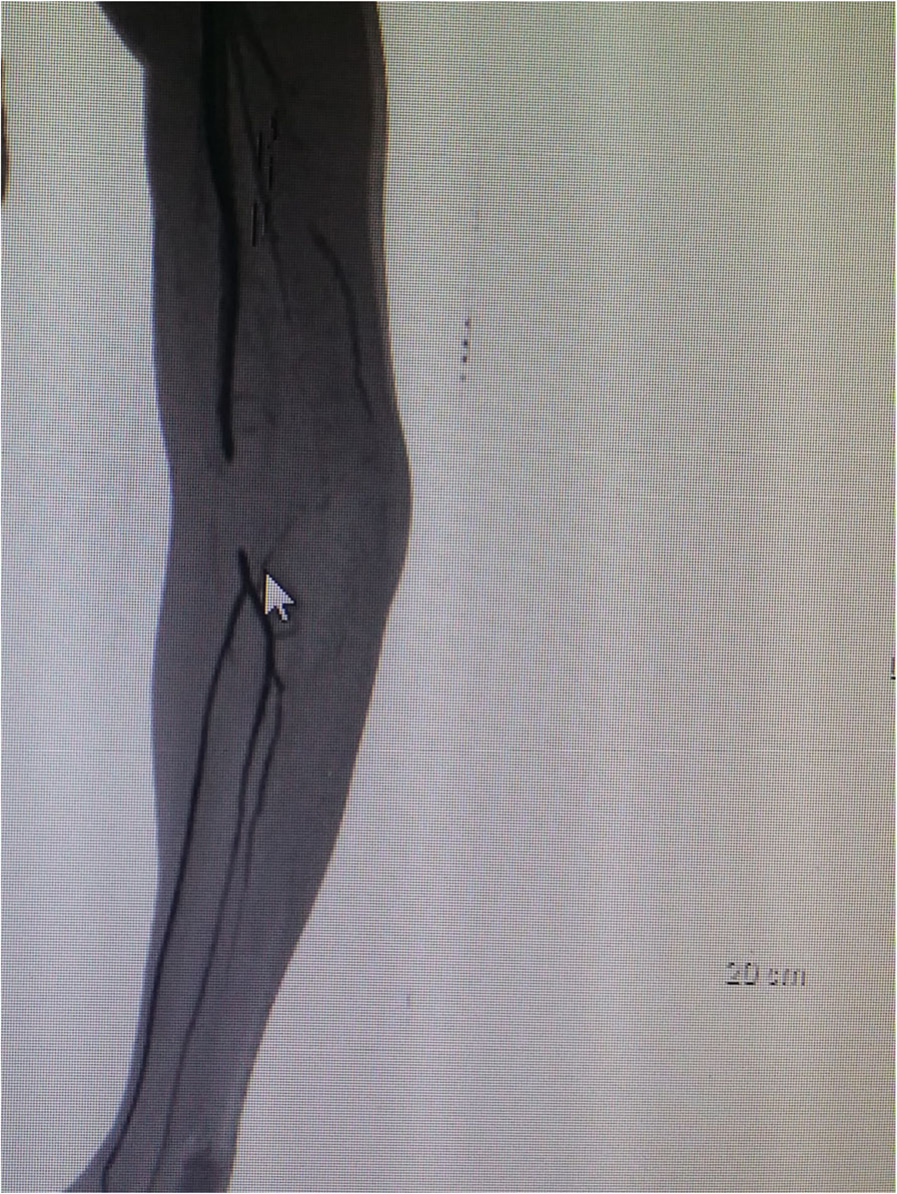
Computed tomography angiogram of left upper limb. Arrow indicating non opacification of brachial artery on CTA

**Fig. 4:**
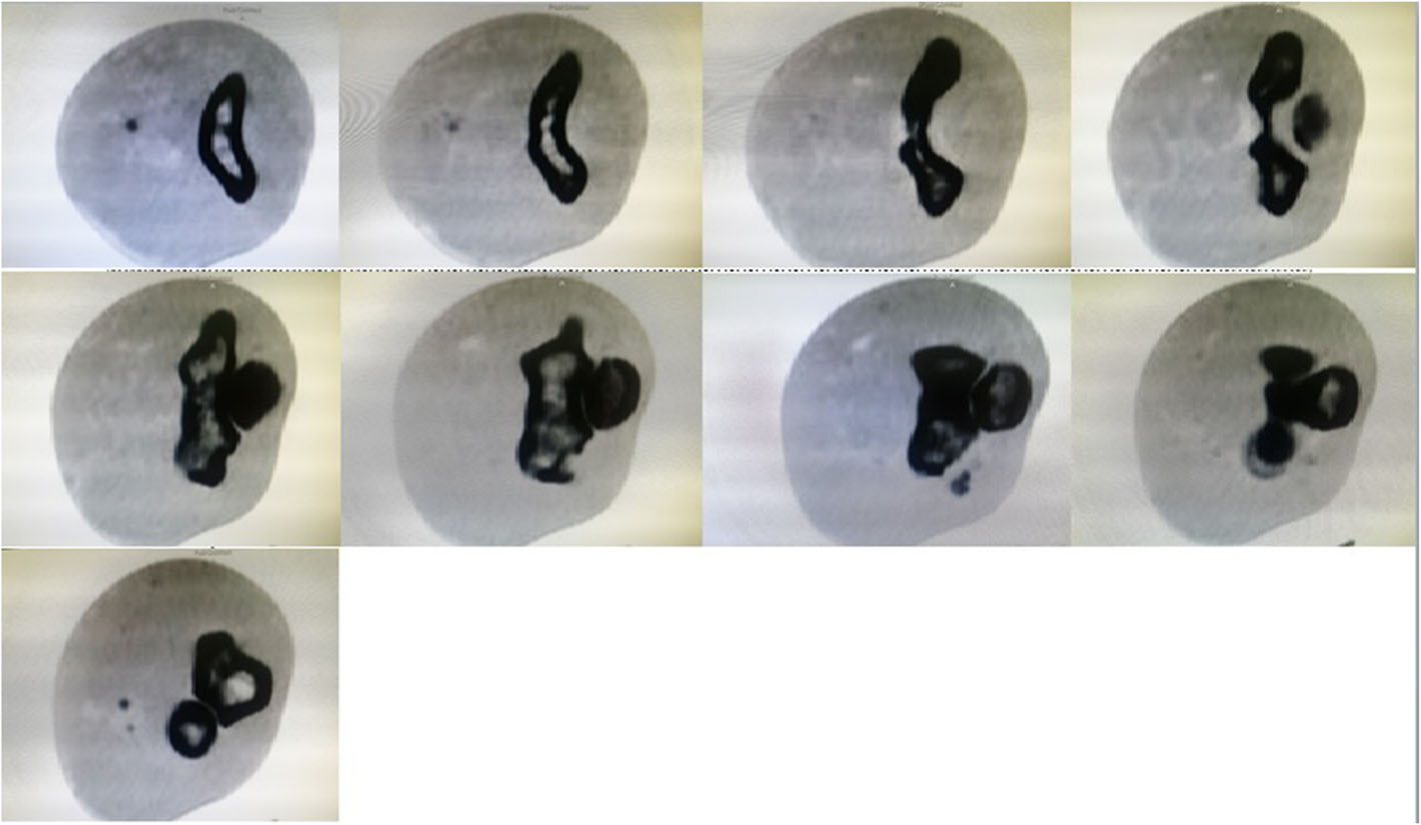
Axial view computed tomography of left elbow

In our Emergency Department, his vital signs were stable with blood pressure 132/80, pulse rate 79/minute, and temperature 37 °C. A physical examination of his left upper limb revealed a tender, fluctuant swelling over the left antecubital fossa with slight limitation in his left elbow range of motion due to pain. There was ecchymosis over the lateral aspect of his left elbow joint but his left elbow was not deformed. His left radial pulse was feeble and his left ulnar pulse was not palpable. Capillary refill times of all fingers were more than 2 seconds. Sensation over left upper limb was normal. Doppler signal of brachial artery proximal to cubital fossa was triphasic, radial artery was monophasic, and ulnar artery was absent. Radiographs of his left elbow showed chip fracture over the left lateral epicondyle of the humerus (Figs. 1 and 2). Subsequently an urgent computed tomography angiogram of his left upper limb was done which showed a segment of non-opacification of contrast at the distal left brachial artery measuring 3.3 cm with distal reconstitution of the left brachial artery by collaterals just before the bifurcation of the left brachial artery at the left elbow joint (Figs. 3 and 4). The computed tomography scan also showed minor fractures of left lateral epicondyle and left radial head (Fig. 4). Laboratory investigations (full blood count and renal function test) were all normal.

He was seen by general surgery and orthopedics teams. Our hospital did not have vascular expertise; hence, he was referred and transferred to a vascular surgeon in another hospital for surgery. He underwent emergency left brachial artery exploration surgery 15 hours after his fall. On intraoperative examination, his distal left brachial artery was contused. Therefore, a left brachial to brachial artery bypass was done using reversed saphenous vein graft. Intravenously administered antibiotics (cefuroxime 750 mg three times a day) were given before induction and for 3 days postoperatively. Postoperatively, Doppler signals of left radial and ulnar arteries had improved. He did not develop reperfusion syndrome requiring fasciotomy. The vascular repair was successful and he was discharged 4 days after surgery. On discharge, his bilateral radial pulses were symmetrical and strong. Fractures over left lateral epicondyle and left radial head were treated conservatively using a 90 degrees posterior splint for 2 weeks. The plan was to immobilize these fractures for a short duration followed by early range of motion exercises.

**Fig. 5:**
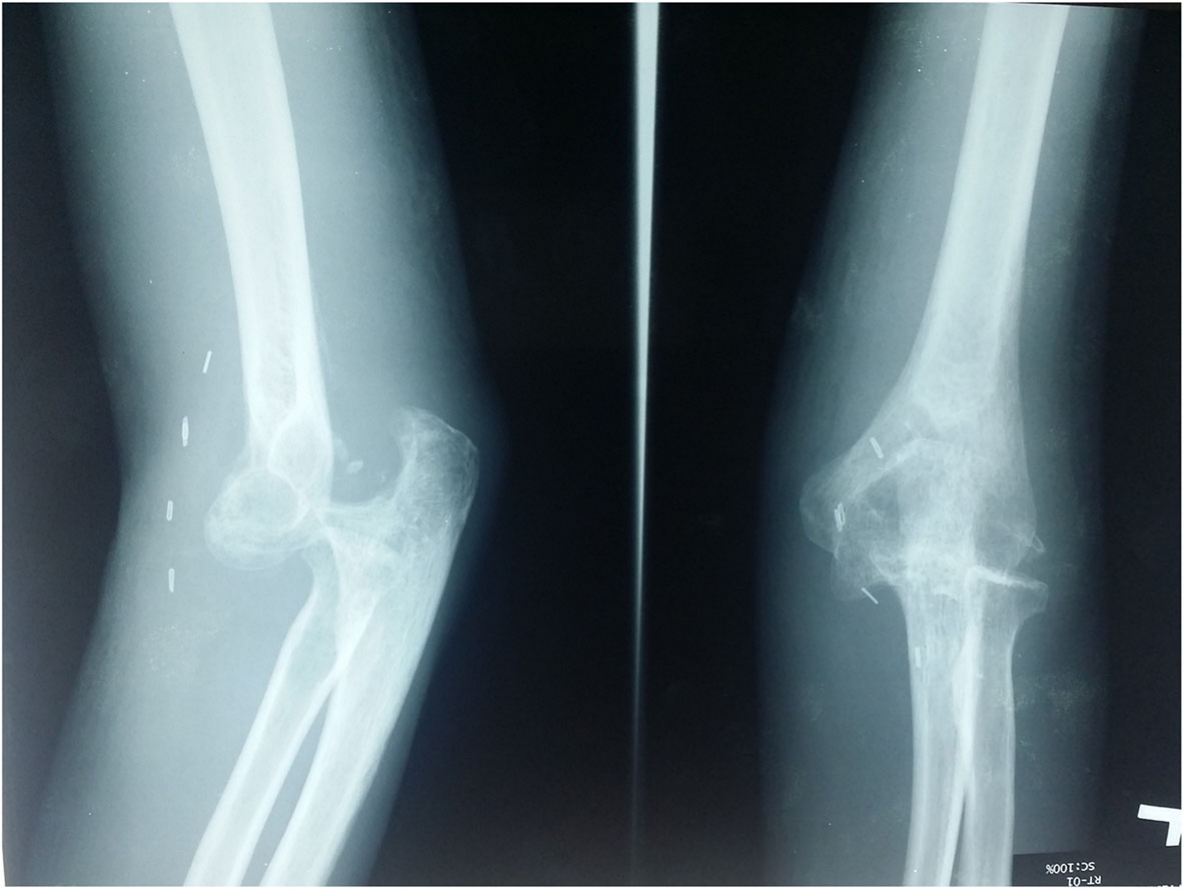
Radiograph of left elbow before closed manipulative reduction

**Fig. 6:**
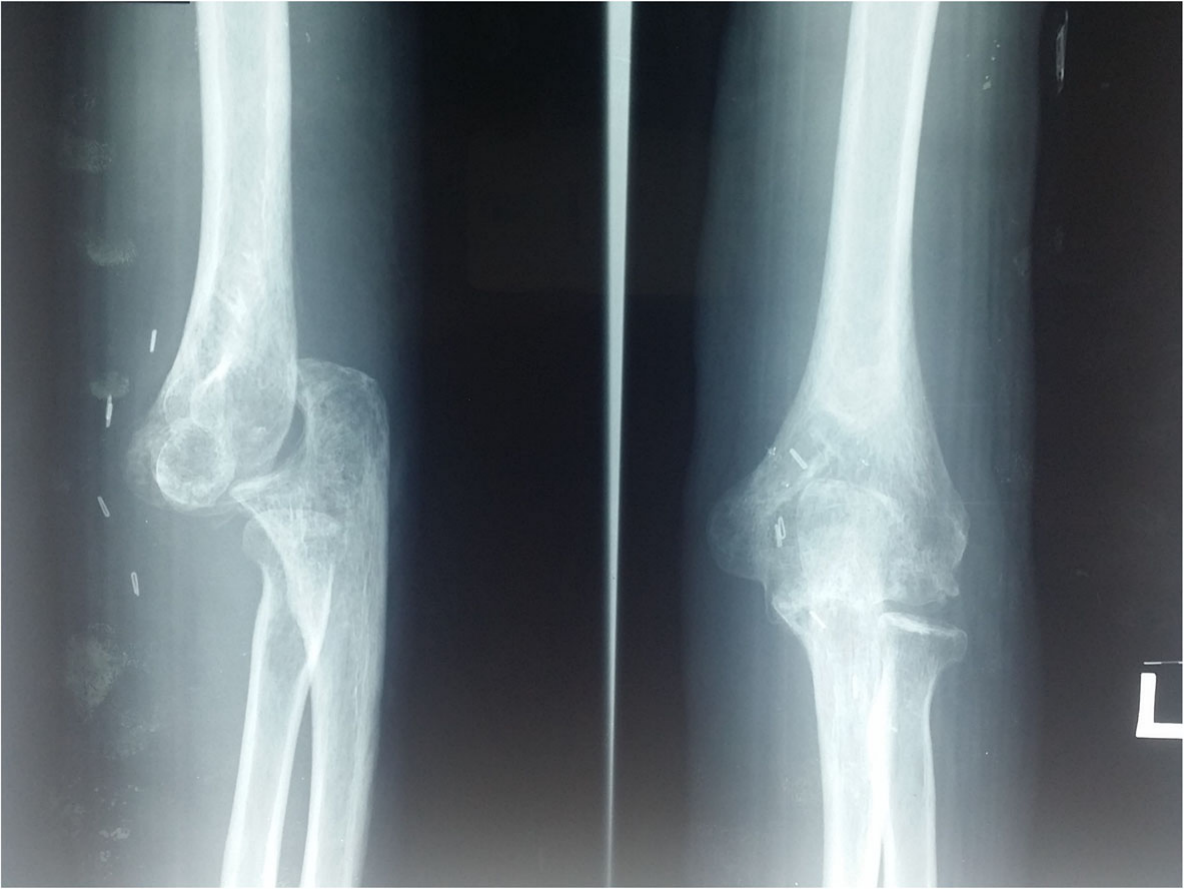
Radiograph of left elbow after closed manipulative reduction

This patient was followed up in orthopedic and vascular out-patient clinics. Six weeks post trauma, his left elbow was noted to be dislocated in an out-patient clinic (Fig. 5). Closed manipulative reduction was attempted but unsuccessful. His left elbow was still subluxed (Fig. 6). There was probably soft tissue interposition in the left elbow joint. His left upper limb neurovascular examination was intact. He was counselled for surgery to reduce the elbow joint with vascular team standby. However, he was not keen for surgery at that time. At the last clinic follow-up around 6 months post trauma, his left elbow joint was still subluxed, his left triceps was shortened, and left elbow range of motion was reduced (extension 0 degrees, flexion 45 degrees, and pronation and supination normal). His radial pulses were strong and equal bilaterally. Functionally, he was able to cope with light duties. He used his left shoulder to compensate for the reduced range of motion of his left elbow. However, he was unable to carry weight > 2 kg using his left upper limb. He was still not keen for any surgical intervention to stabilize his elbow joint due to the risk of vascular graft thrombosis and injury.

## Discussion

Our case report describes a middle-age patient with 20 pack year smoking history who sustained blunt trauma causing brachial artery injury, minor fractures around the elbow joint, and occult elbow instability/dislocation not evident clinically and radiologically at initial presentation.

The brachial artery is one of the commonest arteries injured in the upper limb [3]. The mechanism and common site of brachial artery injury can be explained by relating to the regional anatomy of the upper limb. The brachial artery is a continuation of the axillary artery which runs in the anterior compartment of the arm down to the medial aspect of the antecubital fossa where it bifurcates into the radial and ulna artery. Many brachial artery injuries occur at the antecubital fossa proximal to the bifurcation of the brachial artery [2]. This may be related to vascular compression of the brachial artery as it runs under the lacertus fibrosus at the antecubital fossa [2]. The relative immobility of this artery prevents any longitudinal movement to compensate for external forces to the elbow joint [2].

Blunt trauma causing brachial artery injury is very rare [6–8]. The distal pulses may even be present in the event of brachial artery injury because the elbow joint has a circumferential network of collaterals which can supply the interosseous artery and the recurrent branches of radial and ulnar arteries [2]. Historically, ligation of brachial artery was done to control post-traumatic bleeding at surgery without compromise to distal circulation [9]. In contrast to predictions, our case study showed that thrombosis of the brachial artery may cause ischemia of the hand. Even though there is evidence of reconstitution of blood flow in the forearm on angiography, the blood flow is not strong enough to produce a positive signal on pulse oximetry, good Doppler sounds, and palpable radial and ulnar pulses. The absence of pulse oximetry signal on the fingers is a strong indication of inadequate tissue perfusion. The presence or absence of distal pulses after thrombosis of brachial artery could be explained by an anatomical variant of the collateral circulation [9]. Our patient’s tobacco smoking history might also contribute to poor distal circulation. Some studies have also found that distal ischemia is also related to the degree of damage to the collateral circulation around the elbow joint at the time of injury [9]. The current trend in management of brachial artery injury caused by blunt trauma with evidence of distal ischemia is surgical revascularization using reversed saphenous vein graft [6, 9, 10].

The incidence of posterior elbow dislocation causing brachial artery injury reported in one multicenter trial was 0.47% [8]. In rare cases, elbow dislocation may not be evident clinically or radiologically [11, 12]. In our case study, imaging studies did not show any significant dislocation. Therefore a high index of suspicion is required to diagnose subtle elbow instability post trauma. Our patient had elbow dislocation with associated fracture of lateral epicondyle and radial head. This is classified as complex elbow dislocation. Several studies have suggested ligament reconstruction, cross elbow external fixator, or transarticular pin fixation at the time of vascular repair, whereas other authors immobilize the elbow using posterior splint for varying durations [2, 6, 9–11, 13, 14]. There is no clear consensus regarding the method and duration of immobilization for elbow dislocation associated with brachial artery injury. Most of these studies reported on simple elbow dislocation without any associated elbow fracture [6, 9, 13]. The majority of these studies have shown good functional outcome by employing different treatment plans [2, 10, 15]. However, the patient in our study developed elbow dislocation 6 weeks post vascular repair with suboptimal functional outcome. Fortunately, after a follow-up for 6 months, the elbow instability did not cause any significant vascular compromise.

Our case outcome is different to another similar case of occult elbow dislocation reported by McMurtry and Bhullar who immobilized the elbow joint using a posterior splint for 3 weeks but managed to regain good functional outcome of the elbow joint [11]. Again, McMurtry and Bhullar’s patient sustained simple elbow dislocation without associated fractures [11]. One study has shown the outcome of complex elbow dislocation is poorer than simple elbow dislocation [16]. We postulated that in a case of elbow dislocation with minor fractures around the elbow joint associated with brachial artery injury, immobilization using a 90 degree posterior splint for 2 weeks is not a sufficient treatment. The gold standard management for complex elbow dislocation associated with brachial artery injury in the acute setting is not well reported in the literature. Hence, further studies on the management of complex elbow dislocations associated with vascular injury will be beneficial to provide more insight on the best way and duration of immobilization of the elbow joint to achieve better functional outcome.

## Conclusions

This is an uncommon case of brachial artery injury in a civilian caused by blunt trauma associated with occult elbow instability/dislocation and minor fractures around the elbow joint. The treatment of brachial artery injury with clinical evidence of distal ischemia is surgical revascularization. The possibility of elbow instability and dislocation need to be considered in all cases of brachial artery injury because early radiograph and computed tomography scans may be normal. Short-term posterior splint immobilization is not sufficient to prevent recurrent dislocation.

## Authors’ information

MFBM and SBI are practicing orthopedic surgeons in Hospital Sultan Ismail Johor Bahru Malaysia.

ZHP is a second-year trainee in Masters of Orthopedic Surgery.
